# Characterization of *P. vivax* blood stage transcriptomes from field isolates reveals similarities among infections and complex gene isoforms

**DOI:** 10.1038/s41598-017-07275-9

**Published:** 2017-08-10

**Authors:** Adam Kim, Jean Popovici, Amélie Vantaux, Reingsey Samreth, Sophalai Bin, Saorin Kim, Camille Roesch, Li Liang, Huw Davies, Philip Felgner, Sócrates Herrera, Myriam Arévalo-Herrera, Didier Ménard, David Serre

**Affiliations:** 1 Institute for Genome Sciences, University of Maryland, Baltimore, Maryland USA; 2 Malaria Molecular Epidemiology Unit, Institut Pasteur in Cambodia, Phnom Penh, Cambodia; 3 Division of Infectious Diseases, University of California Irvine, Irvine, California USA; 4 Caucaseco Scientific Research Center, Cali, Colombia; 5 School of Health, University of Valle, Cali, Colombia; 6 Unité Biologie des Interactions Hôte-Parasite, Institut Pasteur, Paris, France; 7 Inserm U1016, CNRS UMR8104, Institut Cochin, Paris, France

## Abstract

Our understanding of the structure and regulation of *Plasmodium vivax* genes is limited by our inability to grow the parasites in long-term *in vitro* cultures. Most *P. vivax* studies must therefore rely on patient samples, which typically display a low proportion of parasites and asynchronous parasites. Here, we present stranded RNA-seq data generated directly from a small volume of blood from three Cambodian vivax malaria patients collected before treatment. Our analyses show surprising similarities of the parasite gene expression patterns across infections, despite extensive variations in parasite stage proportion. These similarities contrast with the unique gene expression patterns observed in sporozoites isolated from salivary glands of infected Colombian mosquitoes. Our analyses also indicate that more than 10% of *P. vivax* genes encode multiple, often undescribed, protein-coding sequences, potentially increasing the diversity of proteins synthesized by blood stage parasites. These data also greatly improve the annotations of *P. vivax* gene untranslated regions, providing an important resource for future studies of specific genes.

## Introduction

*Plasmodium vivax* is the second largest cause of human malaria around the world, accounting for about 8.5 million cases in 2015 and almost half of the reported malaria infections outside of sub-Saharan Africa^1^. Most strategies deployed to eliminate malaria primarily target falciparum malaria and are less effective in controlling vivax malaria, the frequency of which is increasing in many endemic regions^2^. Basic research on *P. vivax* has greatly fallen behind studies of *P. falciparum* due to a lack of continuous *in vitro* culture system. Studies of *P. vivax* often depend on clinical samples and are complicated by the parasite genetic diversity, the polyclonality of many infections, as well as the host genetic diversity and the confounding effects of previous exposures. Genomic techniques, including whole genome sequencing, have provided new tools for understanding *P. vivax* biology, but have so far only modestly improved our understanding of the biology of this pathogen^3–5^. In particular, *P. vivax* genes are still incompletely annotated and the regulation of the parasite genes expressed, even during blood stage infections, remains poorly understood^2^.

Most studies of gene expression in *Plasmodium* parasites have been conducted using *P. falciparum*, due to its public health importance and its ability to be grown *in vitro*, which i) facilitates acquisition of study material, ii) enables synchronization of the parasite stages, and iii) provides a controlled (though artificial) environment. Fortunately, many of the observations initially made in *P. falciparum* have later been validated in other *Plasmodium* species^6^. For example, the patterns of gene expression throughout the intraerythrocytic cycle of rodent parasites *in vivo* are very similar to those of *P. falciparum in vitro* ^7^. *Plasmodium* mRNAs also show conserved features across species, such as longer 5′ and 3′ untranslated regions (UTRs) than observed in most eukaryotes^8–10^. Transcription of noncoding RNAs is also conserved^7, 9, 11–16^ with the presence of snRNAs, that facilitate intron removal by the spliceosome, and snoRNAs, that are required for rRNA processing, methylation, and pseudouridylation (reviewed in ref. 6). While investigations in *P. falciparum* are essential for characterizing fundamental mechanisms of gene regulation of *Plasmodium* parasites, they are unlikely to be sufficient for understanding specific biological features of other human malaria parasites (which are only distantly related to *P. falciparum*). For example, *P. vivax* only invades young reticulocytes, and infections typically lead to much lower parasitemia than *P. falciparum* infections^17, 18^. It is therefore essential to complement *P. falciparum* studies with direct analyses of the other human malaria parasites, including *P. vivax*, to ultimately eliminate malaria worldwide.

Here, we describe for the first time, analyses of *P. vivax* transcriptomes directly generated from 50 uL of capillary blood collected from three Cambodian vivax malaria patients. We showed that by using globin and ribosomal RNA depletion prior to library preparation, we were able to remove sufficient host RNA to thoroughly characterize the parasite transcriptome. Using stranded RNA-seq, we *de novo* assembled the *P. vivax* transcripts of each clinical infection and compared them to each other and to the reference annotation. Our analyses showed that the blood stage *P. vivax* transcriptome is similar between infections despite differences in the proportion of their parasite stages. Additionally, we were able to thoroughly characterize individual transcripts and their 5′- and 3′-UTRs, noncoding RNAs, and potentially novel protein isoforms throughout the genome. Finally, we compared the gene expression profiles of blood stage parasites with those of sporozoites to further expand our understanding of *P. vivax* genes and their regulation.

## Results

### Ribosomal and globin RNA depletion enables comprehensive characterization of *P. vivax* transcriptomes from clinical blood samples

To characterize the diversity of RNA molecules expressed by *P. vivax* parasites during clinical infections, we analyzed stranded RNA-seq libraries prepared from three patients. For each patient, we extracted ~200 ng of total RNA from approximately 50 uL of whole blood. We removed host ribosomal RNAs (rRNAs) and globin mRNAs using magnetic beads before preparing strand-specific RNA-seq libraries^19^. We then generated more than 50 million paired-end reads of 50 bp for each of the samples (Table 1). We aligned the reads successively to the human (Hg38) and *P. vivax* (PlasmoDB-29) reference genomes taking into account the strand information kept during the library preparation (see Materials and Methods). 63.7–77.7% of reads mapped to the human genome, but less than 1% of these reads aligned to globin genes and host rRNA genes. Overall, 16.0–30.4% of reads mapped to the *P. vivax* genome, resulting in more than 10 million paired-end reads from each infection (Table 1).

**Table 1:** Summary statistics of the infections and genomic analyses for the three blood stage samples (V_DJK_8, V_DJK_10, V_DJK_16) and the sporozoite sample (SP_1).

	Blood sample 1	Blood sample 2	Blood sample 3	Salivary gland sample
	V_DJK_8	V_DJK_10	V_DJK_16	SP_1
Parasite density (parasites/µL)	750	6.970	11.000	—
Parasite-stages proportion (thick/thin blood films)				
Ring	22%	79%	35%	—
Trophozoite	45%	14%	65%	—
Schizont	0%	0%	0%	—
Gametocyte	33%	7%	0%	—
**Mapping**				
#Read pairs generated	65,219,377	55,945,558	69,641,135	437,202,828
#Read pairs mapped to human (%)	50,647,696 (77.66%)	35,653,630 (63.73%)	53,537,602 (76.88%)	—
#Read pairs mapped to human, duplicates removed (%)	24,607,961 (48.59%)	21,835,168 (61.24%)	40,479,261 (74.61%)	—
Read pairs mapped to rRNAs (%)	9,878 (0.04%)	7017 (0.03%)	9106 (0.02%)	—
Read pairs mapped to globin mRNAs (%)	59,327 (0.24%)	19624 (0.09%)	22117 (0.05%)	—
Reads mapped to other annotated protein-coding genes (%)	16,291,255 (66.20%)	16,463,840 (75.40%)	23,090137 (57.04%)	—
#Reads pairs mapped to P. vivax (%)	10,436,776 (16.00%)	16,988,674 (30.37%)	11,208,385 (16.09%)	17,833,896 (4.08%)
#Read pairs mapped to P. vivax, duplicates removed (%)	3,778,226 (36.20%)	8,828,288 (51.97%)	7,249,998 (64.68%)	1,594,798 (8.94%)
Read pairs mapped to rRNAs (%)	1,880 (0.5%)	2,717 (0.03%)	2,167 (0.3%)	230 (0.01%)
Reads mapped to annotated protein-coding genes (%)	1,668,417 (44.16%)	3,983,570 (45.12%)	3,729,411 (51.44%)	984,997 (61.8%)
**Individual** ***de novo*** **assembly**				
#Read pairs used for Trinity	4,080,296	9,211,092	7,532,645	2,650,203
#Transcripts assembled (% reads)	15,746 (75.30%)	21,477 (96.25%)	20,631 (96.99%)	7,359 (57.74%)
#Transcripts expressed >10X (% reads)	4,298 (68.82%)	9,516 (92.47%)	8,654 (92.87%)	6,221 (57.52%)
noncoding transcripts (% reads)	1,471 (31.95%)	2,990 (44.68%)	2,708 (38.99%)	4,146 (18.17%)
partial protein-coding transcripts (% reads)	1,642 (11.88%)	3,848 (16.28%)	3,454 (17.36%)	1,866 (25.35%)
complete protein-coding transcripts (% reads)	1,185 (24.98%)	2,678 (31.51%)	2,492 (36.51%)	209 (14.00%)
encoding unique AA sequences	1017	2235	2029	187
assembled in combined Trinity	893 (87.8%)	1875 (83.9%)	1697 (83.6%)	—
#Transcripts single position (% reads)	15421	20781	20049	7311
**Combined** ***de novo*** **assembly**				
#Read pairs used for Trinity	20,824,238			
#Transcripts assembled (% reads)	29,510 (95.28%)			
#Transcripts expressed >10X (% reads)	15,951 (93.68%)			
noncoding transcripts (% reads)	6,348 (41.02%)			
partial protein-coding transcripts (% reads)	5,762 (17.45%)			
complete protein-coding transcripts (% reads)	3,841 (35.21%)			
encoding unique AA sequences	3044			

One of the advantages of RNA-seq experiments is that they provide a comprehensive perspective on all transcripts expressed and not only well-characterized genes (as opposed to, for example, gene expression microarrays). In addition, in our experiments, we removed rRNAs before library preparation and thus avoided poly-A selection, which enabled us to characterize all RNAs expressed, not only polyadenylated ones. As a consequence, only 44.2–51.4% of the reads aligned to the *P. vivax* genome mapped within annotated protein-coding genes (and on the same strand) (Table 1). We ruled out that this low proportion of reads mapped to *P. vivax* protein-coding genes was caused by DNA contamination during our library preparation (Supplemental Fig. 1).

### *De novo* transcript assembly confirms the protein-coding annotations of most *P. vivax* genes

To obtain an unbiased perspective of the *P. vivax* blood stage transcriptome, we *de novo* assembled the RNA transcripts produced by the parasites in each patient infection. We assembled a total of 15,746–21,477 putative *P. vivax* transcripts per patient (Table 1). Most of the putative RNA molecules (93.5–95.9%) mapped to a single location in the *P. vivax* reference and only 4.1–6.5% of the transcripts mapped partially to two different places in the genome, possibly representing chimeras generated during *de novo* assembly. We then focused on highly expressed transcripts (>10 X on average) that are more likely to have been fully assembled (rather than representing fragments of incompletely assembled transcripts). For all further analyses, we therefore concentrated on 4,298–9,516 transcripts (27.3–44.3% of the initial assembled transcripts) accounting for 68–93% of all reads that aligned to the *P. vivax* genome (Table 1).

We predicted that 2,827–6,526 of these highly-expressed transcripts (65.8–68.7%) encoded for proteins of more than 100 amino acids. These included 1,185–2,678 putative full-length protein-coding genes that encoded both a start and a stop codon. 1,642–3,848 additional transcripts lacked a start codon, a stop codon, or both; and likely represented portions of protein-coding genes that were not fully assembled into a complete transcript (Table 1). These potentially truncated transcripts were typically shorter and had lower read coverage than the full-length transcripts (Supplemental Fig. 2) and could possibly be entirely reconstructed with additional sequencing data.

The 1,185–2,678 highly expressed, complete protein-coding transcripts represented 1,017–2,235 unique amino acid sequences (see discussion of isoforms below). 914–1,890 of these protein sequences (89.9–84.6%) matched an annotated *P. vivax* protein sequence with more than 90% identity over more than 90% of their length (out of 5,552 protein-coding genes annotated in the *P. vivax* genome). In addition, for 74–282 protein sequences (7.3–12.6%), the similarity was greater than 90% but only over a portion of the amino acid sequence (>50%) suggesting that the transcript reconstructed was either a protein isoform of the annotated gene or, possibly, that the current annotation was partially incorrect (see also below). Finally, 58–147 predicted amino acid sequences did not match any annotated *P. vivax* protein sequence (see below for more discussion).

### The blood stage transcriptomes generated from different patient infections are remarkably similar

**Figure 1:**
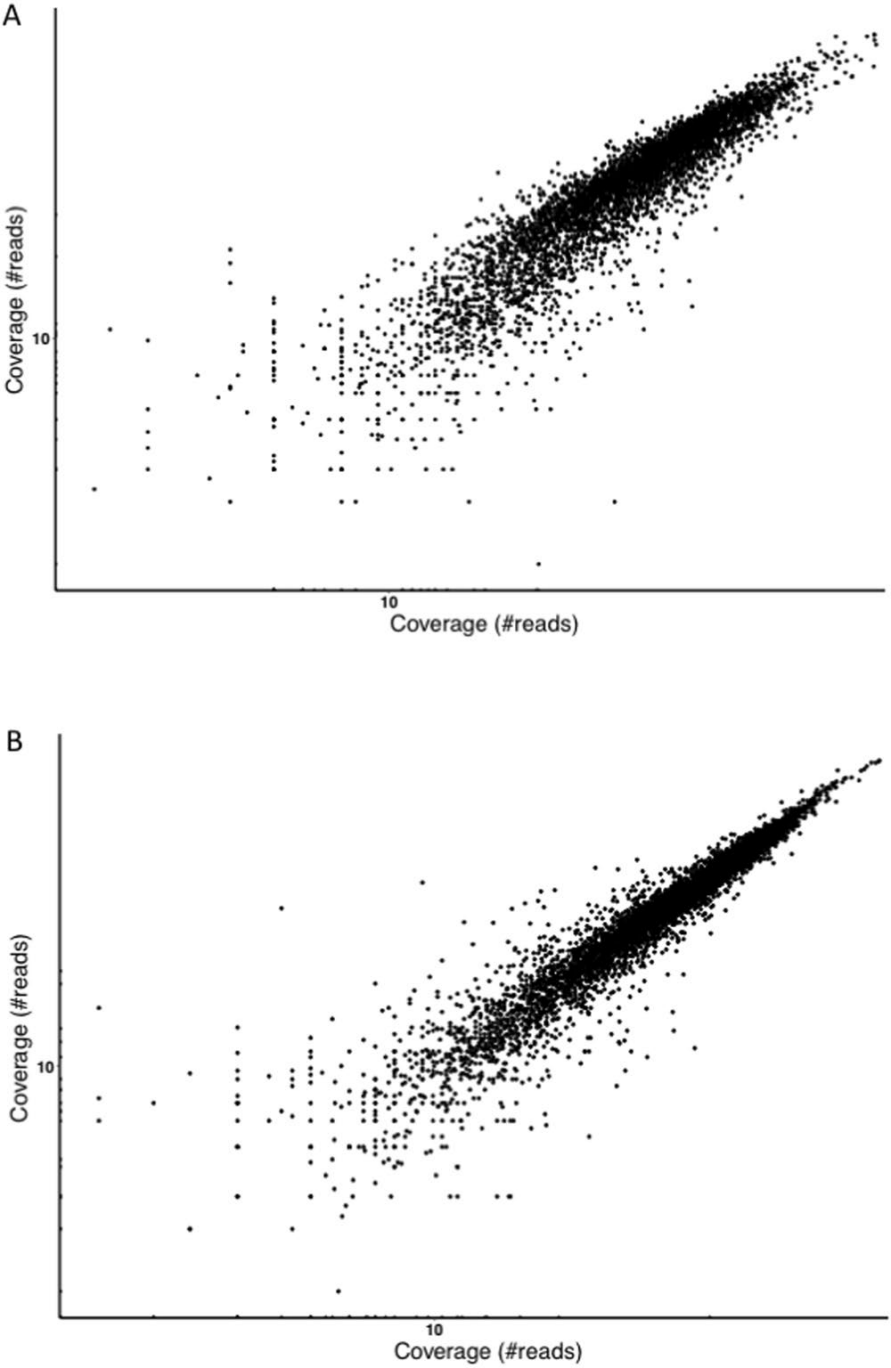
Correlation between the parasite gene expression patterns in two infections. The figure shows the number of reads mapped to each annotated gene (black dots) in the RNA-seq data generated from **(A)** the infection of patient V_DJK_8 (x-axis) and patient V_DJK_16 (y-axis) and (**B**) the infection of patient V_DJK_10 (x-axis) and patient V_DJK_16 (y-axis).

*P. vivax*-infected blood samples typically contain a mixture of different developmental stages, each with their own specific gene expression patterns^9^. The three clinical infections analyzed here showed extensive variations in the relative proportions of parasite stages: at the time of collection, one infection was primarily composed of ring stage parasites, a second predominantly consisted of late trophozoites, and the third included a high proportion of gametocytes (Table 1). We therefore tested how different were the patterns of gene expression generated from each of the three patient infections. First, we looked at the most abundant parasite transcripts in each infection. We observed a large overlap among samples, with, for example, 16 genes being present among the 25 most expressed genes of each sample (Table 2) and 75 common genes among the top 100 genes (Supplemental Table 1). The similarity in expression pattern was observed throughout the entire transcriptome and the patterns of gene expression in one infection were, overall, largely conserved across infections (Fig. 1, r^2^ > 0.8, p-value < 2.2 × 10^−16^), despite their differences in parasite stage composition. When we considered genes that are believed to be specifically expressed at a given developmental stage, the differences in gene expression were similarly not obvious, except for the ookinete surface antigen precursor gene (PVX_111175, the ortholog of Pfs25) that was significantly more highly expressed in the clinical infection with a high proportion of gametocytes (Supplemental Table 2).

**Table 2:** List of the 25 most expressed genes in each sample (ranked by their relative coverage in read counts per bp).

V_DJK_8_0			V_DJK_10_0			V_DJK_16_0			Sp_1		
Gene ID	Name	Cov.	Gene ID	Name	Cov.	Gene ID	Name	Cov.	Gene ID	Name	Cov.
PVX_092995	tryptophan-rich antigen (Pv-fam-a)	34.39	PVX_117322	glyceraldehyde-3-phosphate dehydrogenase putative	63.06	PVX_117322	glyceraldehyde-3-phosphate dehydrogenase putative	55.51	PVX_001715	early transcribed membrane protein (ETRAMP)	17.86
PVX_003565	early transcribed membrane protein (ETRAMP)	31.09	PVX_003565	early transcribed membrane protein (ETRAMP)	45.54	PVX_003565	early transcribed membrane protein (ETRAMP)	42.13	PVX_123510	cell traversal protein for ookinetes and sporozoites	7.83
PVX_117322	glyceraldehyde-3-phosphate dehydrogenase putative	21.06	PVX_000010	Plasmodium exported protein unknown function	42.45	PVX_000010	Plasmodium exported protein unknown function	40.06	PVX_089425	heat shock 70 kDa protein putative	3.53
PVX_000010	Plasmodium exported protein unknown function	20.98	PVX_114830	elongation factor 1-alpha putative	30.20	PVX_090930	histone H4 putative	31.70	PVX_088870	early transcribed membrane protein (ETRAMP)	2.48
PVX_097583	skeleton-binding protein 1 putative	19.89	PVX_114832	elongation factor 1-alpha putative	28.31	PVX_114015	histone H2A putative	30.97	PVX_122910	hypothetical protein conserved	2.29
PVX_096020	Plasmodium exported protein unknown function	19.34	PVX_090930	histone H4 putative	27.93	PVX_083045	phosphoethanolamine N-methyltransferase	25.61	PVX_091975	hypothetical protein conserved	2.09
PVX_093680	Phist protein (Pf-fam-b)	19.02	PVX_095015	enolase putative	27.18	PVX_095015	enolase putative	24.54	PVX_099035	inhibitor of cysteine proteases putative	1.68
PVX_112670	unspecified product	16.62	PVX_114015	histone H2A putative	26.75	PVX_114830	elongation factor 1-alpha putative	24.47	PVX_119355	circumsporozoite (CS) protein	1.65
PVX_090930	histone H4 putative	16.20	PVX_093680	Phist protein (Pf-fam-b)	22.81	PVX_090935	histone 2B	24.37	PVX_096315	hypothetical protein conserved	1.45
PVX_114830	elongation factor 1-alpha putative	14.67	PVX_119470	40 S ribosomal protein S23 putative	21.53	PVX_097583	skeleton-binding protein 1 putative	23.49	PVX_080040	hypothetical protein conserved	1.44
PVX_113235	Pv-fam-d protein	13.87	PVX_090935	histone 2B	20.88	PVX_114832	elongation factor 1-alpha putative	23.24	PVX_093500	gamete release protein putative	1.39
PVX_114832	elongation factor 1-alpha putative	13.54	PVX_097583	skeleton-binding protein 1 putative	20.66	PVX_093680	Phist protein (Pf-fam-b)	20.55	PVX_118040	gamete egress and sporozoite traversal protein putative	1.28
PVX_101520	Pv-fam-d protein	13.42	PVX_092820	60S ribosomal protein L41 putative	19.58	PVX_113235	Pv-fam-d protein	19.28	PVX_087935	DNA-directed RNA polymerase II 8.2 kDa polypeptide putative	1.25
PVX_112665	unspecified product	13.10	PVX_113235	Pv-fam-d protein	18.88	PVX_119470	40S ribosomal protein S23 putative	19.12	PVX_117605	thioredoxin 1 putative	1.24
PVX_114015	histone H2A putative	12.08	PVX_123060	DNA/RNA-binding protein Alba 1 putative	18.74	PVX_092820	60S ribosomal protein L41 putative	17.83	PVX_100695	CHCH domain containing protein	1.22
PVX_090935	histone 2B	11.65	PVX_087860	60S ribosomal protein L37 putative	17.76	PVX_003955	60S ribosomal protein L37a putative	17.55	PVX_099860	hypothetical protein	1.18
PVX_101595	Plasmodium exported protein unknown function	11.61	PVX_080245	40S ribosomal protein S9 putative	17.36	PVX_087860	60S ribosomal protein L37 putative	17.51	PVX_082735	thrombospondin-related anonymous protein	1.18
PVX_119470	40S ribosomal protein S23 putative	10.64	PVX_003955	60S ribosomal protein L37a putative	17.11	PVX_087825	40S ribosomal protein S29 putative	17.30	PVX_122540	hypothetical protein conserved	1.14
PVX_096035	hypothetical protein	9.78	PVX_113725a	60S ribosomal protein L39 putative	16.75	PVX_092995	tryptophan-rich antigen (Pv-fam-a)	17.04	PVX_000810	perforin-like protein 1	1.10
PVX_123060	DNA/RNA-binding protein Alba 1 putative	9.32	PVX_089425	heat shock 70 kDa protein putative	16.54	PVX_113665	histone H3 putative	16.87	PVX_122458	conserved Plasmodium protein unknown function	1.09
PVX_092820	60S ribosomal protein L41 putative	9.27	PVX_119587	60S acidic ribosomal protein P2 putative	16.48	PVX_101520	Pv-fam-d protein	16.77	PVX_117755	nifU protein putative	1.09
PVX_115470	Pv-fam-d protein	8.17	PVX_087825	40S ribosomal protein S29 putative	16.43	PVX_113725a	60S ribosomal protein L39 putative	16.10	PVX_001015	6-cysteine protein putative	1.06
PVX_113725a	60S ribosomal protein L39 putative	8.14	PVX_099535	phosphoglycerate kinase putative	16.17	PVX_080245	40S ribosomal protein S9 putative	15.94	PVX_098795	hypothetical protein	1.05
PVX_087825	40S ribosomal protein S29 putative	8.13	PVX_116630	lactate dehydrogenase	15.55	PVX_119587	60S acidic ribosomal protein P2 putative	15.01	PVX_118255	fructose 1,6-bisphosphate aldolase putative	1.03
PVX_087860	60S ribosomal protein L37 putative	8.08	PVX_091640	phosphoglycerate mutase putative	15.33	PVX_112670	unspecified product	14.78	PVX_002900	secreted protein with altered thrombospondin repeat domain putative	0.99

### *P. vivax* genes may encode different protein isoforms

We next focused on characterizing potential protein isoforms (i.e., different protein variants encoded by the same gene) expressed by blood stage parasites. Since our analyses showed little variation among samples, we combined the reads generated from all three patients to increase the read coverage and performed a new *de novo* transcript assembly resulting in 29,510 transcripts, with 15,951 having greater than 10 X average coverage. Note that this “combined” assembly recapitulated more than 83% of the transcripts observed in each individual assembly (Table 1). Similar to the numbers obtained in the individual assemblies, these transcripts included: 3,841 predicted full-length protein-coding transcripts (24.1%), 5,762 partial protein-coding transcripts (36.1%) and 6,348 noncoding (39.8%). We mapped all full-length protein-coding transcripts to the *P. vivax* reference genome. Out of the 3,841 full-length protein-coding transcripts, 611 transcripts (15.9%) had an amino acid sequence identical to that of another transcript and mapped to the same location. For the analyses of protein isoforms, we discarded these transcripts and focused on the 3,230 unique protein-coding sequences.

2,298 potentially protein-coding transcripts (71.1%) were the sole transcribed product of a gene: no transcript with a different predicted protein-coding sequence mapped to the same location, and we therefore did not observe any evidence of variations in amino acid sequences for these genes (Supplemental Fig. 2). For 1,955 of these transcripts (85.1%), the predicted protein sequences were more than 90% similar to the annotated amino acid sequences. 15 transcripts (0.7%) had only partial sequence identity (>50%) suggesting that the most expressed isoform in blood stage parasites differed from the annotated isoform or that the current annotation was partially incorrect. Finally, 328 transcripts (14.3%) mapped to regions of the genome with no annotated protein-coding genes and may represent novel genes. However, most of these transcripts had very short predicted coding sequences (80% were shorted than 150 amino acids) and likely include false positives (the complete list of these transcripts and their annotations is presented in Supplemental Table 3).

**Figure 2:**
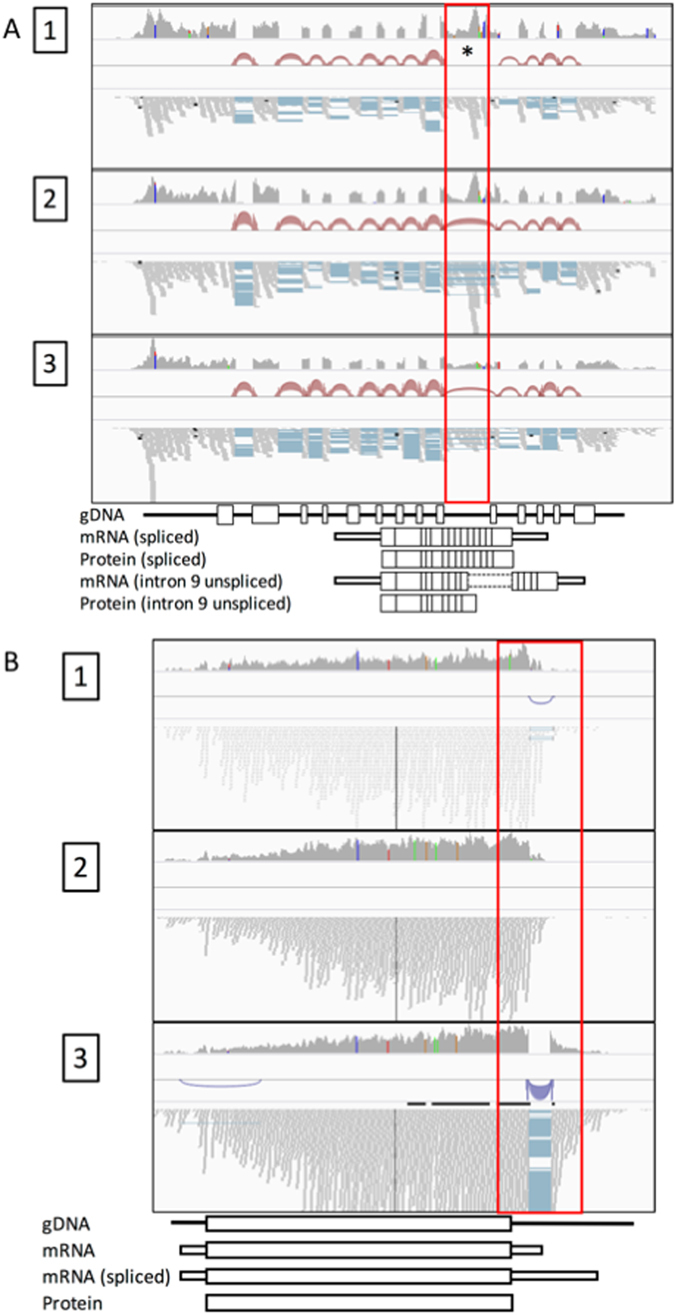
Examples of transcripts differing from the current *P. vivax* gene annotations. Data from each patient are displayed in successive rows (labeled 1–3). For each data set, the grey track shows the read coverage, the bridges display evidence of intron splicing and the blue and red bars the actual reads generated. (**A**) Read coverage across PvCRT. Note that intron 9 is retained in some of the transcripts from infections 2 and 3 and in all transcripts from infection 1 (red box). (**B**) Read coverage across PvMDR1. Note that in two infections, some PvMDR1 transcripts contains an unannotated intron in 3′-UTR resulting in a longer transcript (red box).

The remaining 932 transcripts (28.9%) represent protein isoforms of 412 predicted genes, with some genes expressing up to eight different predicted amino acid sequences (Supplemental Fig. 3A). These multiple isoforms were responsible for the vast majority of the transcripts that were only partially similar to the annotated protein sequences and, in 89.2% of the cases (165/185 genes), one of the protein isoforms corresponded perfectly to the PlasmoDB annotation (Supplemental Fig. 3B). Note that the proportion of expressed genes potentially encoding multiple protein isoforms was comparable in the combined (14.6%) and individual assemblies (6.6–10.6%), and that these predicted protein isoforms are therefore unlikely to be artefacts caused by pooling reads from different infections. In addition, only 21 genes, out of the 412 genes with multiple isoforms, belonged to multigene families (including 13 *vir* genes). The remaining 391 genes with multiple isoforms were single copy genes, indicating that most of these multiple isoforms were not caused by misassembly or mismapping of highly similar paralogous DNA sequences but represented genuine cases of alternative transcription. One interesting example of a gene with multiple potential protein isoforms was the chloroquine resistance transporter (PvCRT, PVX_087980), a gene possibly associated with chloroquine resistance. In all three patients analyzed here, the 9^th^ intron was predominantly retained (i.e., unspliced), with one patient showing no evidence of splicing at all (Fig. 2A). This intron retention is predicted to alter the following 13 amino acids before introducing a premature stop codon at position 330 (instead of 427 in the classic annotation of the protein sequence), resulting in a much shorter protein (if translated).

### Most 5′- and 3′-UTRs are incompletely annotated and can vary among isoforms

**Figure 3:**
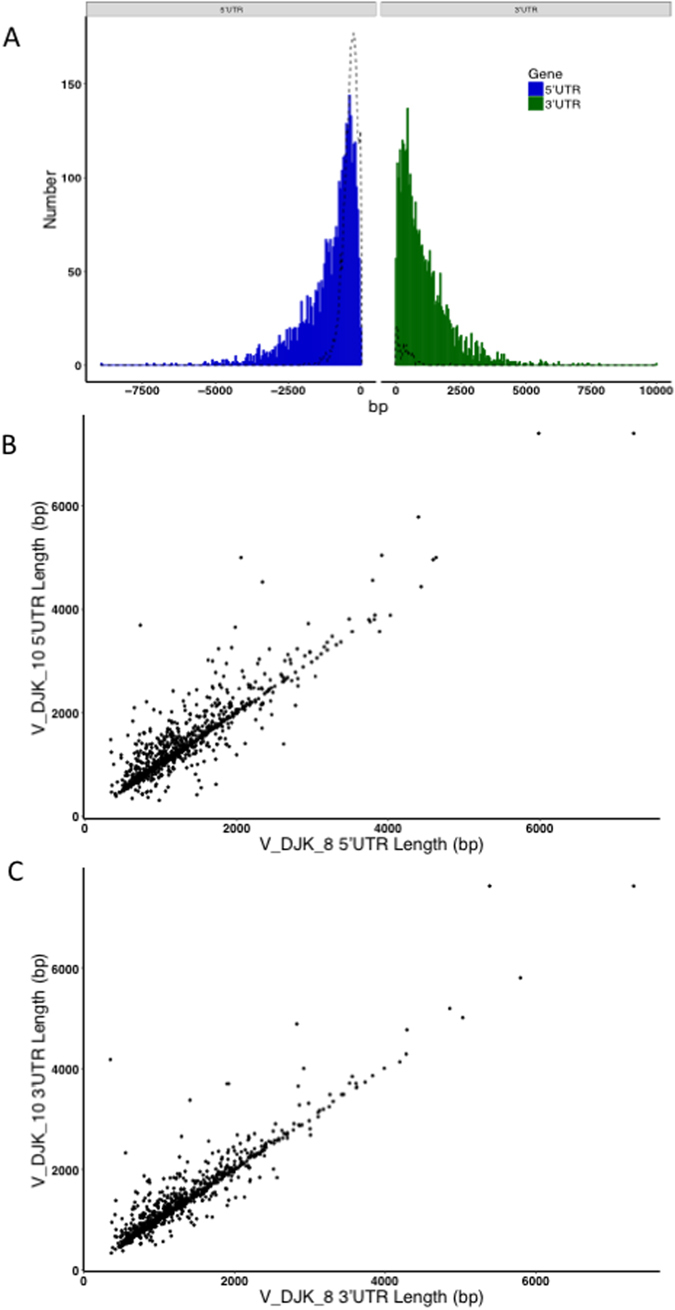
Distribution of the length of untranslated regions for full-length protein-coding transcripts. (**A**) The histogram shows the number of protein-coding transcripts (y-axis) with a given 5′- and 3′-UTR length (x-axis, in blue and green respectively). The dashed line represents the currently annotated UTR length for all *P. vivax* protein-coding genes. (**B**) Pair-wise comparison of 5′UTR lengths between samples V_DJK_10 and V_DJK_8. (**C**) Pair-wise comparison of 3′UTR lengths between samples V_DJK_10 and V_DJK_8. Additional comparisons in Supplemental Figure 4.

Most of the current *P. vivax* gene annotations derive from amino acid sequence prediction and orthology to *P. falciparum*. As a consequence, the untranslated regions (UTRs) of many genes are poorly characterized despite their importance in transcription and mRNA stability. For example, there are only 140 *P. vivax* genes with annotated 3′-UTR in PlasmoDB-29. Our analyses provided a first description of UTRs for 3,230 *P. vivax* transcripts, with a median length of 754 bases and 785 bases for, respectively, 5′- and 3′-UTRs (Fig. 3A). Interestingly, the UTR of the same transcripts determined independently from different individual infections show little variation in length (Fig. 3B,C, Supplemental Figure 4), indicating that these extended UTRs are genuine (though the exact boundaries might not be entirely accurately characterized). Consistent with previous reports in other *Plasmodium* species^9, 16, 20^, we observed the presence of introns in these extended UTRs: 172 transcripts contained one or more introns in their 5′-UTR while 74 transcripts contained one or more introns in their 3′-UTR.

A number of *de novo* assembled transcripts had identical predicted protein-coding sequences (Table 1), and we therefore looked in more details at these isoforms to determine if they were caused by variations in UTR lengths. Among the 412 genes expressing multiple isoforms with the same predicted amino acid sequences, 147 genes contained alternative promoter start sites (i.e., difference in 5′-UTR), 99 genes contained alternative termination sites (i.e., difference in 3′-UTR), and 32 genes had transcripts with varying 5′- and 3′-UTRs (Supplemental Figure 5). One example of isoforms generated by variation in UTR length is the Multi-Drug Resistance gene 1 (PvMDR1, PVX_080100), also a candidate marker for chloroquine resistance, for which some transcripts (encoding an identical amino acid sequence) displayed splicing of an unannotated 3′-UTR intron, resulting in a longer UTR (Fig. 2B).

### Noncoding RNAs in *P. vivax*

Finally, we analyzed the 6,348 transcripts with no evidence of protein-coding potential (i.e., with an ORF smaller than 100 amino acids). While the coding transcripts varied greatly in size, with a median size of ~1,856 bases, the noncoding transcripts displayed a much narrower distribution, with a median length of ~458 bases and 88.5% of the noncoding transcripts smaller than 1,000 bases (Supplemental Fig. 2A). This smaller size is likely influenced by our criteria for defining protein-coding genes (>100 amino acids) and could thus be biased by the inclusion of fragments of protein-coding transcripts. Note however that a few of these noncoding RNAs were much longer, with 205 noncoding transcripts greater than 1,500 nucleotides.

These noncoding transcripts included five known *P. vivax* snRNAs and several rRNAs (two 5.8S, three 18S and three 28S rRNA), which accounted for 28.4% of all reads generated from *P. vivax* RNAs (Table 1). The other 6,321 transcripts did not appear to be related to any noncoding RNAs previously characterized in *Plasmodium*. Interestingly, 685 (10.8%) of these transcripts contained introns, a phenomenon not well understood for noncoding RNAs but that has been described in other malaria parasites^21^.

We then analyzed the origin of these noncoding transcripts with regards to protein-coding genes (Supplemental Figure 6). 134 of the noncoding transcripts (2.1%) derived from the introns of annotated protein-coding genes. These transcripts could either have been co-transcribed with protein-coding mRNAs and not degraded after splicing, or may have been independently transcribed from a separate promoter. 569 noncoding transcripts (9.0%) aligned to annotated protein-coding genes in the genome, but on the opposite strand (Supplemental Figure 6). Antisense transcripts (i.e., RNAs synthesized from the opposite strand of a protein-coding gene) have been shown to regulate the transcription of the protein coding gene^13^. Interestingly, while antisense RNAs have been shown to regulate the expression of *var* genes in *P. falciparum* ^22^, we did not observe any antisense transcription in *vir* gene clusters, suggesting that *P. vivax vir* genes might be regulated differently than *P. falciparum var* genes.

### Comparison of blood stage and sporozoite transcriptomes

We generated one RNA-seq library using RNA obtained from sporozoites isolated from the salivary glands of infected mosquitoes. Since the depletion used for removing human rRNA from blood samples is unlikely to efficiently work for *Anopheles* rRNAs, we used poly-A selection prior to library preparation (and therefore only analyzed polyadenylated transcripts). Out of 437 million read pairs generated, 16.7 million reads (3.8%) aligned to the *P. vivax* genome sequence (Table 1). Using the same approach as described above, we then *de novo* assembled these reads into 7,348 transcripts, including 6,221 with >10X average coverage (Table 1). Of these, only 198 transcripts (3.4%) were predicted to encode a full-length protein, and these transcripts accounted for 14% of the reads mapped to the *P. vivax* genome. 1,866 additional transcripts (30.0%) represented partial protein-coding genes (i.e., missing a start codon, a stop codon, or both) while the remaining 4,146 transcripts (66.6%) did not contain any ORF of more than 100 amino acids and were categorized as putative noncoding RNAs (Table 1). Note that the *de novo* transcripts assembled from sporozoites only accounted for 57.5% of all reads mapped to *P. vivax*, compared to greater than 92% of reads for 2 of the 3 patient infections and 68.9% of reads for the last infection (Table 1). This observation indicated that we might not have enough sequences to characterize the sporozoite transcriptome to the same extent as in blood stage parasites and that we probably failed to assemble a significant fraction of the transcripts expressed at this stage.

Given this limitation, it is interesting to note that 81 of the 198 highly expressed protein-coding transcripts in sporozoites (40.9%) were not detected in blood stage parasites. In fact, the most highly expressed genes differed significantly between blood stage parasites and sporozoites. In blood stage parasites, the most expressed genes were typical housekeeping genes, such as ribosome associated proteins and histones, while in sporozoites, cell invasion genes, like perforin or thrombospondin, were among the most highly expressed (Table 2). We also failed to fully assemble transcripts from most housekeeping genes in sporozoites as well as any *vir* genes or *Pv-fam* genes, which were also abundantly expressed by blood stage parasites.

Since we used poly-A selection before generating the sequencing library, our data is highly biased towards poly-adenylated RNAs. However, we did identify ten transcripts corresponding to rRNAs, specifically, ribosomal RNAs known to be expressed in sporozoites^23^. No small RNAs (snRNA, snoRNA, or tRNAs) were assembled.

Development of protein microarrays enabled comprehensive assessments of the antibody responses to *P. vivax* proteins. For example, a recent study identified 280 highly reactive *P. vivax* peptides in sera from residents of hypoendemic Peruvian Amazon^24^. These reactive antigens were significantly enriched in genes highly expressed in the patient infections: for example, among the 100 genes most expressed by *P. vivax* blood stage parasites, we observed four times as many reactive antigens as what we would expect solely by chance (Supplemental Figure 7). Overall, 101 of the 280 highly reactive peptides (36.1%) were highly expressed by blood stage parasites (Supplemental Table 3). Five peptides (1.8%) were expressed highly by both sporozoites and blood stage parasites. Finally, 9 peptides originated from transcripts only detected in sporozoites while the remaining 165 were of unknown origin (Supplemental Table 4).

## Discussion

Despite its public health importance and its increased recognition as a major challenge for malaria elimination, research on *P. vivax* has dramatically lagged behind that of *P. falciparum* due to our inability to grow *P. vivax* parasites in culture. In particular, comprehensive studies of *P. vivax* gene expression have been very complicated to implement despite efforts to synthesize gene expression microarrays and attempts to study parasites after short-term culture^9, 25, 26^. Here, we show that it is possible to generate robust transcriptome data from a small volume of capillary blood (~50 uL) collected directly from vivax malaria patients, without any processing of the samples before RNA extraction. The protocol used in this study is easy to implement in the field, as it only requires finger prick blood collection and immediate storage in trizol and could therefore be added to many clinical studies for patients with sufficient parasitemia. Our study showed that depletion of rRNAs and globin mRNAs efficiently removed the vast majority of host RNAs present in blood, sufficiently enriching parasite RNAs to enable direct sequencing. This aspect is critical as it alleviates the need for selecting parasite molecules, which introduces biases in the captured molecules and may miss important but poorly characterized transcripts. Another advantage of this approach is that it circumvents poly-A selection and therefore enables a wider characterization of the mRNAs expressed by the parasites, including many noncoding RNAs that may not be poly-adenylated. In addition, we used stranded library preparation, which preserves the information about the strand of origin of each mRNA molecule sequenced and allows better definition of overlapping transcripts (encoded from opposite strands) and antisense noncoding RNAs.

To obtain an agnostic perspective on the transcripts expressed by blood stage *P. vivax* parasites and avoid ascertainment biases introduced by using a reference annotation, we *de novo* reconstructed the transcripts from three vivax malaria patients. Our analyses revealed the diversity and complexity of the *P. vivax* blood stage transcriptome but also the conservation of the gene expression patterns across infections. While *P. vivax* infections typically display varying proportions of asexual parasite stages, each displaying markedly different gene expression profiles^9^, we observed a striking similarity between the genes expressed in different infections. One hypothesis for explaining this intriguing pattern is that one asexual stage is much more transcriptionally active than the others and, regardless of its actual proportion in the infection, will be responsible for most transcripts, homogenizing the patterns across infections. In our study, one infection (V_DJK_8), had a much higher proportion of gametocytes compared to the other two clinical isolates (Table 1) but, overall, displayed a very similar pattern of gene expression to the other infections (e.g., Fig. 1). Indeed, we did not observe significantly higher expression of genes hypothesized to be transcribed specifically in *P. vivax* gametocytes^26–28^, or homologous to other *Plasmodium* species gametocyte genes^29–32^, with the notable exception of the orthologue to *Pfs25* that was significantly more expressed in the isolate having the highest proportion of gametocytes (Supplemental Table 1).

On the other hand, our study revealed that transcription in *P. vivax* is much more complex than often considered: roughly 10% of all genes expressed in blood stage parasites encoded for more than one amino acid sequence. While it is not clear whether these isoforms eventually lead to different proteins or if they play a role in regulating the transcription and/or translation of these genes, this phenomenon could significantly increase the catalogue of *P. vivax* proteins synthesized. One fascinating example of this phenomenon is PvCRT, a gene possibly involved in chloroquine resistance^33^: for this gene, the most abundant transcript retained the 9^th^ intron unspliced, resulting in a shorter encoded protein, with an alternative C-terminal sequence. This finding illustrates that, even for well-characterized genes, novel isoforms can be discovered and that transcriptomic data could help in deciphering molecular mechanisms responsible for infection or antimalarial drug resistance.

In addition to these potential coding variations, many of the isoforms assembled in our study have an identical predicted protein coding sequence but significantly vary in their 5′- or 3′-UTRs (often due to differential exon splicing). It is possible that *P. vivax* uses different promoters or termination sites to regulate genes transcriptionally and post-transcriptionally. Other groups have noted that UTRs in *Plasmodium* species are longer than in most eukaryotes^9, 16, 21^, but few studies have looked at variations in 5′- and 3′-UTRs and their roles in regulating translation. Our study, by thoroughly characterizing these regions (that were very incompletely annotated previously), also provides an important resource to better investigate the role of these regions and their variability. One gene that would be interesting to further study based on our findings is PvMDR1, which is commonly duplicated in some endemic regions^34^ and has been implicated in chloroquine or mefloquine resistance^35, 36^. Our data clearly indicated the presence of alternatively spliced 3′-UTR introns in some transcripts, and it would be interesting to test whether these isoforms affect PvMDR1 translation or are associated with antimalarial drug resistance.

We also observed a large number of noncoding RNAs transcribed by blood stage parasites. Roughly a third of these RNAs are well-characterized ribonucleoprotein (RNP) forming molecules (e.g., rRNAs, snRNAs). However, we also identified thousands of additional RNAs of unknown function. While it is possible that some of these transcripts are fragments of protein-coding transcripts incompletely assembled in our study, the genomic distribution of these noncoding transcripts, notably in antisense direction of protein-coding genes, suggests that some of them are genuine and possibly play an important role in gene regulation.

Our existing understanding of the molecular mechanisms underlying the biology of *Plasmodium* parasites is essentially derived from studies of *P. falciparum*, and to a lesser extent of rodent parasites. Many observations conducted in these parasites seemed to be generalizable to other *Plasmodium* species. The results of our study are, for example, consistent with previous reports regarding the organization of protein-coding genes and RNAs throughout the genome, the long 5′- and 3′-UTRs and the existence of antisense transcription. However, this pattern might not be universally true and understanding the detailed regulation of a given gene in one species will require direct study of the parasite of interest. For example, we did not find evidence of any of the mechanisms known to regulate *P. falciparum var* genes in *vir* clusters, suggesting that these genes might be regulated differently. The possibility to comprehensively characterize *P. vivax* gene expression patterns directly from patient samples will provide novel opportunities to finely study this hard-to-cultivate parasite and shed light on some of the key biological differences with *P. falciparum*, to eventually improve strategies aiming at better controlling vivax malaria worldwide.

## Method

### Ethical Statement

Informed written consent was obtained for all the participants and the study was approved by the National Ethic Committee at the National Institute of Public Health, Phnom Penh, Cambodia. The methods were performed in accordance with approved guidelines.

### RNA-seq library constructions from vivax malaria patient blood samples

Capillary blood was collected by finger prick from three febrile Cambodian patients seeking antimalarial treatment in health facilities in Ratanakiri province (northeastern Cambodia) in 2014 (Supplementary Figure 8). *P. vivax* mono-infection was confirmed by PCR as described in ref. 37. We determined the parasitemia of each infection using Giemsa-stained thick films and estimated the number of parasites per 200 white blood cells (assuming a white blood cell count of 8000/µL) and the proportion of different parasite stages. 50 uL of blood was preserved immediately in ~500 uL of Trizol and stored at −80 °C. Prior to RNA extraction, the samples were thawed, and brought up to 1 mL with Trizol to account for small variations in the initial amount of blood collected. Total RNA was extracted using the Direct-zol mini-kit according to the manufacturer’s instructions (including an in-column DNase treatment), except that RNA was eluted into 15 uL of DNase/RNase free water. RNA-seq libraries were prepared using the entire volume of purified RNA using the Illumina TruSeq stranded total RNA kit and Ribo-Zero and globin reduction. All three barcoded libraries were pooled together and sequenced on an Illumina HiSeq. 2500 to generate ~65 million paired-end reads of 50 bp for each sample.

### RNA-seq library construction from isolated *P. vivax* sporozoites

To preliminarily characterize the transcriptome of the *P. vivax* sporozoites, we collected 50,000 sporozoites by salivary gland dissections of infected Colombian *Anopheles albimanus* mosquitoes and immediately stored them in RNAlater. We extracted RNA using Qiazol and the Direct-zol mini-kit according to manufacturer’s specifications, resulting in approximately 100 ng of total RNA. We then prepared an RNA-seq library using the Illumina TruSeq stranded mRNA kit with poly-A selection and sequenced it on an Illumina HiSeq. 2500 to generate a total of 437 million paired-end reads of 125 bp.

### Read alignment against the host and parasite genomes

All reads generated from the patient samples were first aligned onto the human reference genome (NCBI Hg38 assembly) using Tophat (version 2.0.9) (Supplementary Figure 9). We then aligned all reads that did not align to the human genome to the *P. vivax* reference genome (PlasmoDB-29) with the following options: -g 1 (to randomly choose a single location for multiple mapped reads), -I 5000 (to only consider introns shorter than 5,000 bases), and -library-type fr-firststrand (to specify mapping of stranded libraries). We then removed all potential PCR duplicates using the samtools rmdup. While this approach might bias estimates of coverage for a few very highly expressed genes, we considered it essential for removing artefacts introduced by the low amount of starting material. We then used custom Perl scripts to calculate read coverage for all annotated exons using the most recent genome annotations of the human (Hg38) and *P. vivax* (PlasmoDB-29) genomes. All sporozoite reads were aligned directly to the *P. vivax* reference genome and processed with the same parameters as described above.

### *De nov*o transcript assembly

We used all non-duplicated reads that mapped to the *P. vivax* reference genome to *de novo* assemble transcripts with Trinity^38^ (version 2.1.1) using the stranded library option (–SS_lib_type RF). Each sample was processed and analyzed independently. All *de novo* assembled transcripts were then mapped to the *P. vivax* PlasmoDB reference genome with GMAP^39^ using -*k 13* (a kmer of 13), *-n 0* (chimeric transcripts are given two paths) and *-f sampe* (paired-end read data). In a separate analysis, we combined all non-duplicated reads from all three clinical infections in a single dataset and *de novo* assembled transcripts using the same parameters as above.

We calculated the read coverage for each transcript, taking into account the strand information: we first separated + and – encoded transcripts based on the GMAP output as well as + or – strand-originating reads based on the initial Tophat mapping. We then used Bowtie2 to align + strand read pairs to + strand transcripts, and - strand read pairs to - strand transcripts. Chimeric transcripts that aligned to two different places in the genome were split and handled as separate transcripts.

### Protein-coding gene predictions

We used Transdecoder^40^ (version 3.0.0) to predict open reading frames (ORFs) from each *de novo* assembled transcript and determine its encoding amino acid sequence. We used the default settings and only considered ORFs encoding for at least 100 amino acids. We further filtered the results and only considered the longest predicted protein-coding sequence of each transcript. Based on these results, we classified each *de novo* transcript into one of three categories: complete protein-coding genes for transcripts containing a start and stop codon, partial protein-coding genes (lacking either a start codon, a stop codon, or both), and noncoding genes (i.e., for transcripts encoding less than 100 amino acids).

### Comparison with current *P. vivax* annotations

To compare the amino acid sequences of the predicted protein-coding genes with the current *P. vivax* protein annotations, we used reciprocal BlastP searches. First, we used BlastP to find, for each predicted protein-coding transcript, the most similar annotated protein(s) in the *P. vivax* reference genome. Then we did the reverse operation to find the most similar predicted protein-coding transcript(s) for each annotated *P. vivax* gene. For both BlastP analyses, we considered amino acid sequences that were more than 90% identical over more than 90% of the length and with an e-value < 0.001. If one transcript and one annotated gene matched each other reciprocally, we classified the predicted protein-coding gene as previously annotated. If one transcript and one annotated gene matched each other only in one search, and not the reciprocal search, but mapped to the same genomic location, we interpreted the predicted protein-coding gene as a protein isoform or a possible misannotation. Finally, if one transcript did not match any annotated protein-coding gene in either search, we classified the transcript as a potentially novel protein-coding gene.

### Annotation of untranslated regions and regulatory isoforms

To identify gene isoforms resulting from differential splicing or alternative 5′ and 3′-UTRs, we began by counting the number of predicted genes that mapped alone to a single location in the genome (i.e., no other transcript mapped to the same location) and defined these as single isoform transcripts. The remaining transcripts (that overlapped with at least one other transcript and were encoded on the same strand) potentially represented evidence of gene isoforms. Since many transcripts aligned to the same location in the genome but only differed by a few nucleotides, we filtered out these redundant transcripts and discarded from further analyses any transcript whose translated products were identical and that displayed 5′- and 3′-ends that did not vary by more than 50 nucleotides. We considered any transcripts mapping to the same location but with different encoding amino acid sequences as potential protein isoforms. To identify variations in 5′- or 3′-UTR, we looked for transcripts with identical protein-coding sequences but differences in 5′- or 3′- ends greater than 50 bp.

### Noncoding RNAs

All transcripts without an ORF of at least 100 amino acids were categorized as noncoding. We used BlastN searches against the entire nt NCBI database to find similarity between these transcripts and annotated rRNA, snRNA, snoRNA and tRNA. We defined antisense noncoding RNAs as transcripts that overlapped known *P. vivax* protein-coding genes over at last 30% of their length but on the opposite strand.

### Data Availability

The sequence data are freely available in NCBI SRA under the BioProjects SUB2480448 and SUB2480498.

## Electronic supplementary material

Supplementary Figures

Supplementary Tables
