# Supplementary material for: Characterization of P. vivax blood stage transcriptomes from field isolates reveals similarities among infections and complex gene isoforms
Source: Sci Rep. 2017 Aug 10;7:7761. doi: 10.1038/s41598-017-07275-9 (PMC5552866; doi:10.1038/s41598-017-07275-9)
Supplement: Supplementary file 1 — Supplementary Figures [file 41598_2017_7275_MOESM1_ESM.pdf]

Characterization of *P. vivax* blood stage transcriptomes from field isolates reveals similarities among infections and complex gene isoforms

Adam Kim[1], Jean Popovici[2], Amélie Vantaux[2], Reingsey Samreth[2], Sophalai Bin[2], Saorin Kim[2], Camille Roesch[2], Li Liang[3], Huw Davies[3], Philip Felgner[3], Sócrates Herrera[4], Myriam Arévalo-Herrera[4,5], Didier Menard[2*] and David Serre[1*]

Supplemental Figure 1. Ruling out DNA contamination. The figure shows the numbers of reads from the RNA-seq experiment from patient V_DJK_10 mapped to the + (x-axis) and – strand (y-axis) of the reference genome sequence. Each blue dot represents a 1 kb window. Note that the coverage in each window is very different for the two strands (in contrast to the 1:1 ratio expected for DNA sequencing).

Supplemental Figure 2. Distribution of the size and read coverage for the *de novo* assembled transcripts. The figure shows the distribution of the transcript length **(A)** and read coverage **(B)** for all *de novo* assembled transcripts according to their coding potential: noncoding transcripts (i.e., less than 100AA) are represented in red, partial protein-coding transcripts (missing either a start or stop codon) in green and full-length protein-coding transcripts in blue.

Supplemental Figure 3. Annotation of full-length protein-coding transcripts. (A) Histogram showing the number of genes (y-axis) that are transcribed into isoforms encoding different amino acid sequences (x-axis). **(B)** The figure shows the proportion of full-length protein-coding transcripts that are identical to the current *P. vivax* protein annotation or differ from it, depending on the number of amino acid encoding isoforms (blue: single amino acid sequence, red: multiple amino acid encoded from the same gene).

Supplemental Figure 4. Pair-wise comparisons of 5'UTR and 3'UTR lengths between each patient sample.

Supplemental Figure 5. Type and numbers of regulatory isoforms identified by de novo assembly.

Supplemental Figure 6. Genomic location of noncoding RNAs with regards to protein-coding genes.

Supplemental Figure 7. Enrichment analysis of reactive antigens among highly expressed blood-stage proteins. All proteins from each infection were averaged and ranked by relative expression. The top 50 proteins with the highest RNAseq counts contain 14 of the reactive 280 antigenic hits by protein microarray[24], resulting in a 5.5 fold enrichment compared to baseline (280 total antigenic hits in 5,477 proteins). Ribosomal proteins were excluded from analysis.

Supplemental Figure 8. Schematic of RNA-seq library preparation.
Blood was drawn from Cambodian patients with vivax malaria. Some of the blood was made into thick smears for calculation of parasite stage and parasitemia. ~50 uL were stored in Qiazol for RNA extraction and preservation. The Direct-zol mini kit was used to extract RNA directly from Qiazol. Then using the Tru-seq globin and mRNA reduction kit, host globin mRNA and ribosomal rRNAs were removed, and libraries were prepared from the rest of the RNA.

Supplemental Figure 9. Schematic of *de novo* assembly.

Figure S1

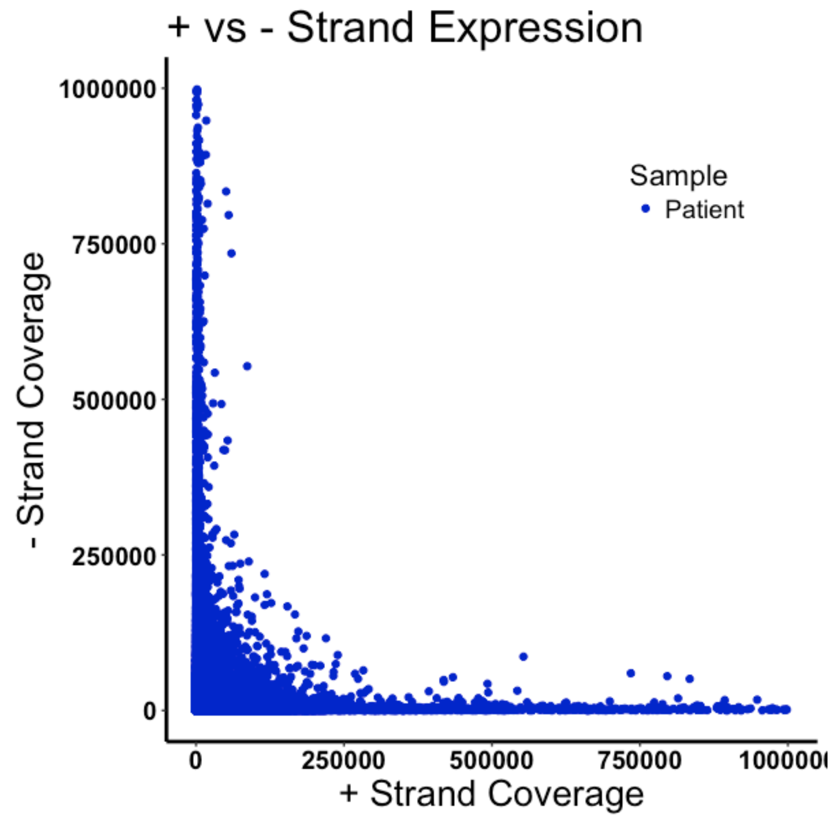
+ vs - Strand Expression
1000000
750000
500000
250000
0
- Strand Coverage
Sample
Patient
0
250000
500000
750000
100000
+ Strand Coverage

Figure S2A

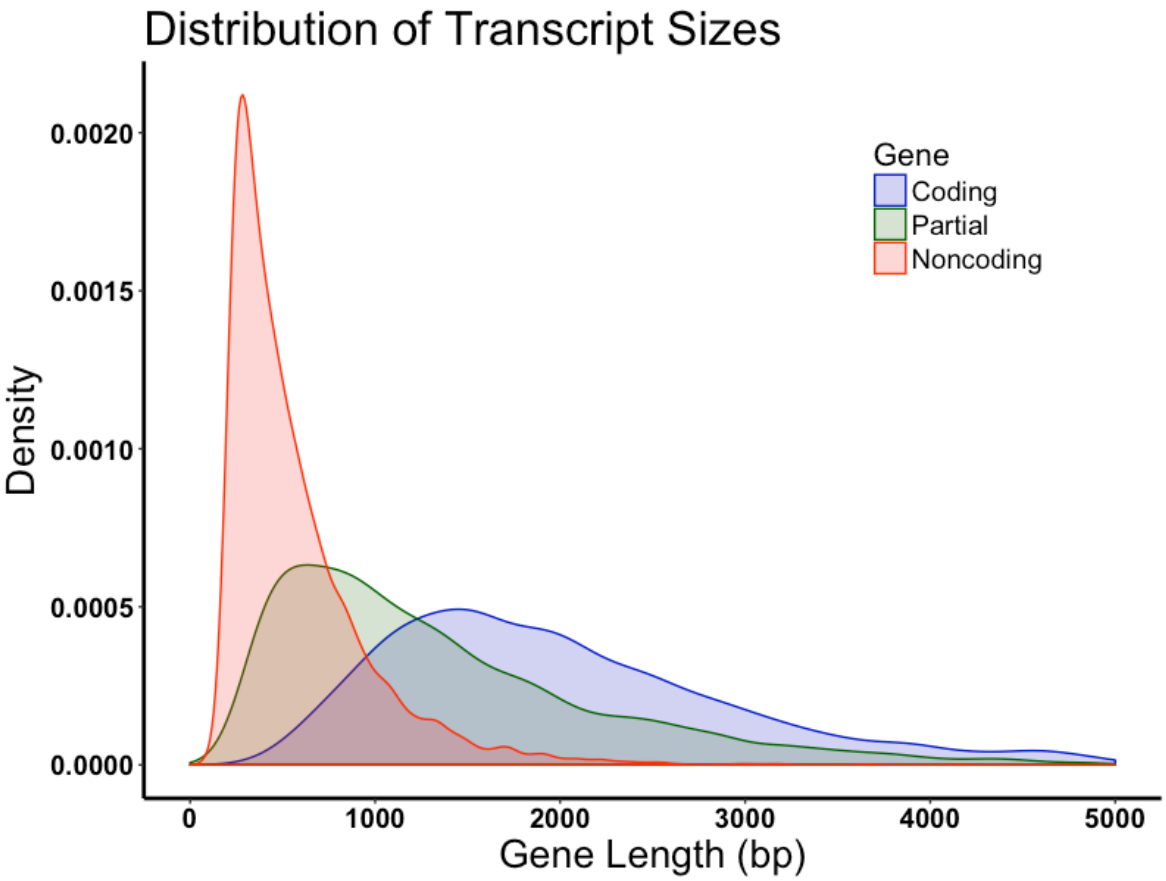

Distribution of Transcript Sizes
Gene
Coding
Partial
Noncoding
Density
0.0020
0.0015
0.0010
0.0005
0.0000
0
1000
2000
3000
4000
5000
Gene Length (bp)

Figure S2B

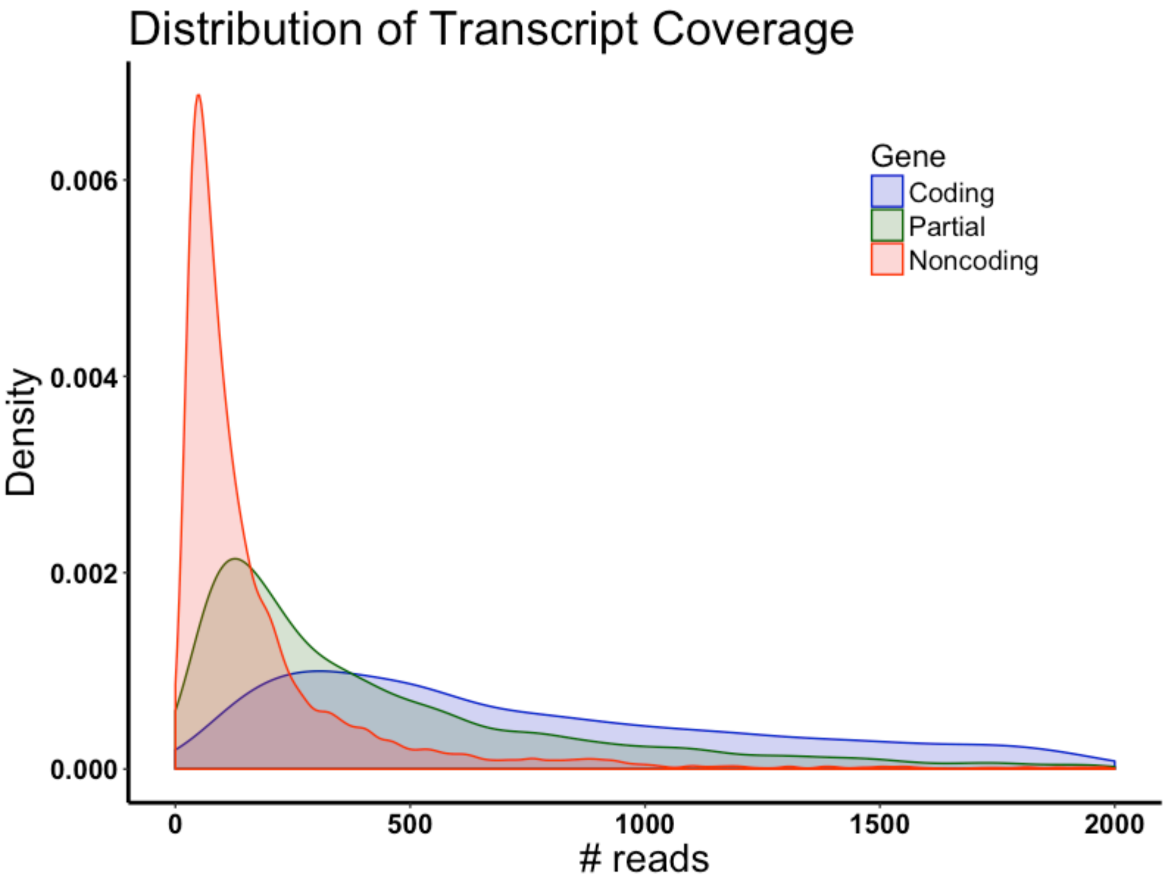
Distribution of Transcript Coverage
Gene
Coding
Partial
Noncoding
Density
0.006
0.004
0.002
0.000
0
500
1000
1500
2000
reads

Figure S3A

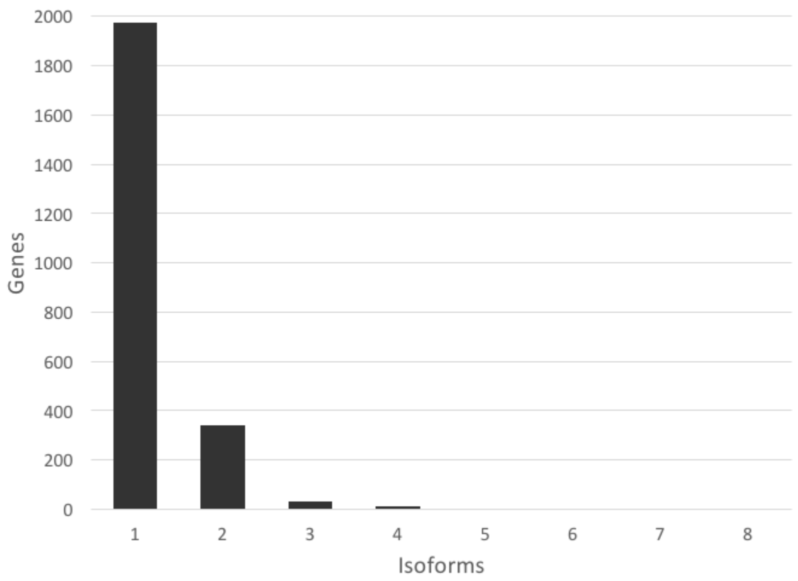

2000
1800
1600
1400
1200
1000
800
600
400
200
0
Genes
1
2
3
4
5
6
7
8
Isoforms

Figure S3B

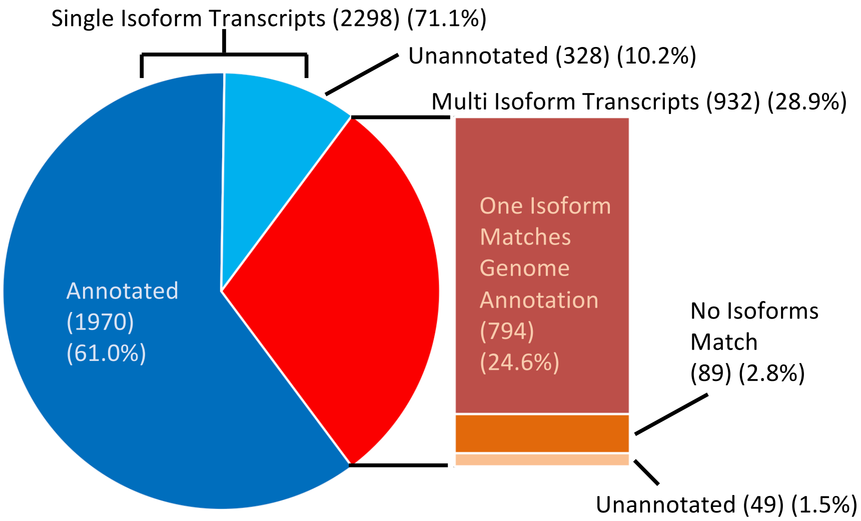

Single Isoform Transcripts (2298) (71.1%)
Unannotated (328) (10.2%)
Multi Isoform Transcripts (932) (28.9%)
Annotated (1970) (61.0%)
One Isoform Matches Genome Annotation (794) (24.6%)
No Isoforms Match (89) (2.8%)
Unannotated (49) (1.5%)

Figure S4

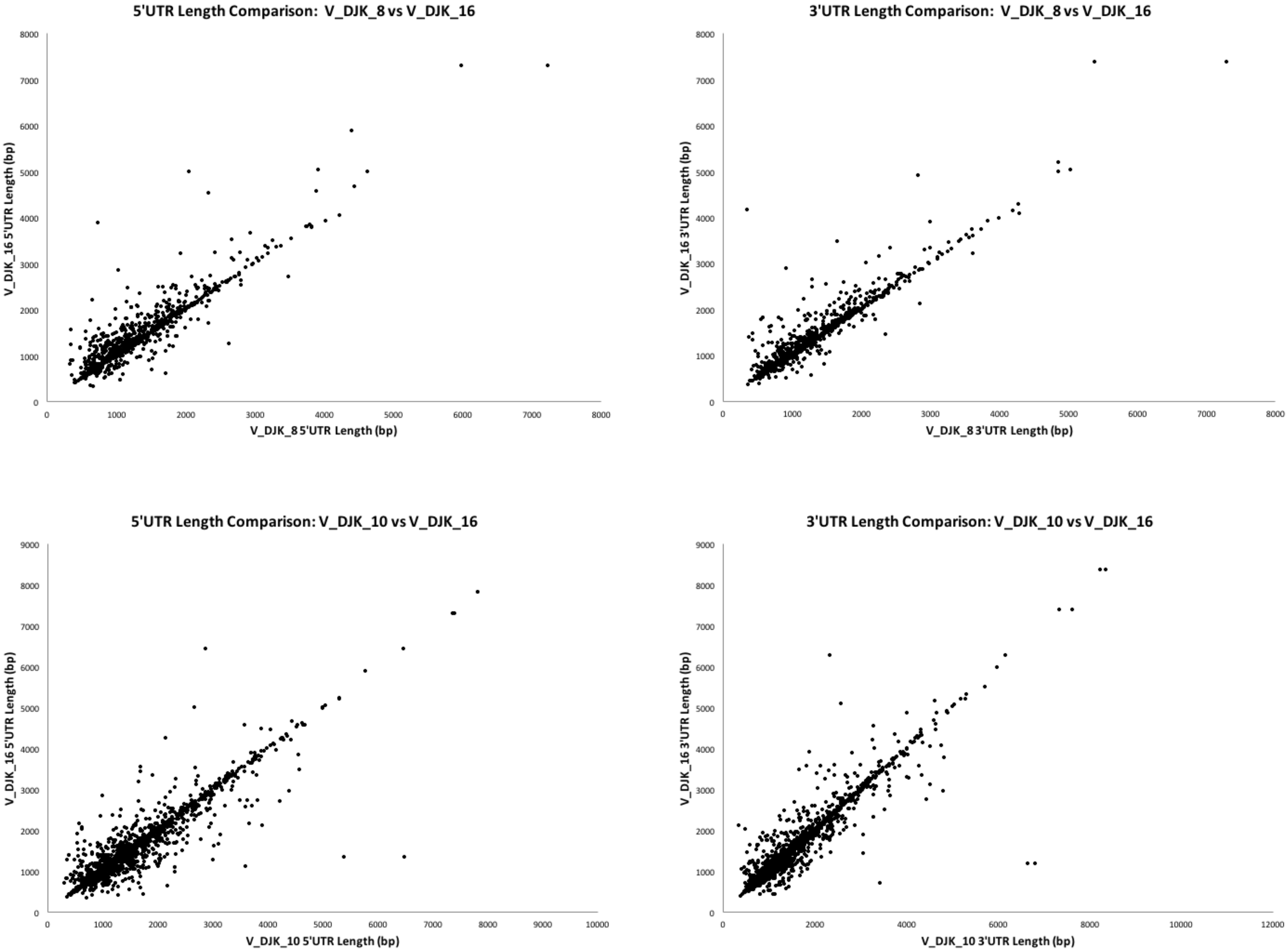

5'UTR Length Comparison: V_DJK_8 vs V_DJK_16
V_DJK_16 5'UTR Length (bp)
V_DJK_8 5'UTR Length (bp)
3'UTR Length Comparison: V_DJK_8 vs V_DJK_16
V_DJK_16 3'UTR Length (bp)
V_DJK_8 3'UTR Length (bp)
5'UTR Length Comparison: V_DJK_10 vs V_DJK_16
V_DJK_16 5'UTR Length (bp)
V_DJK_10 5'UTR Length (bp)
3'UTR Length Comparison: V_DJK_10 vs V_DJK_16
V_DJK_16 3'UTR Length (bp)
V_DJK_10 3'UTR Length (bp)

Figure S5

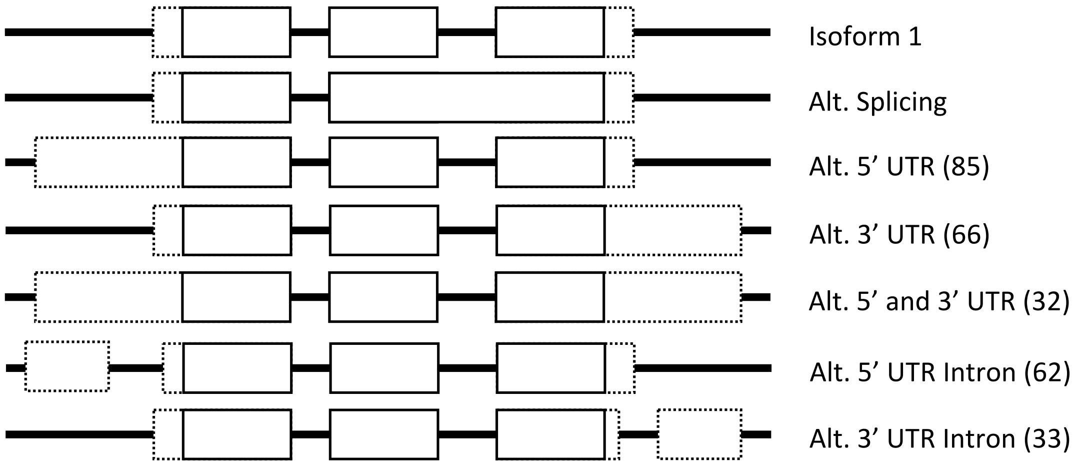
Isoform 1
Alt. Splicing
Alt. 5' UTR (85)
Alt. 3' UTR (66)
Alt. 5' and 3' UTR (32)
Alt. 5' UTR Intron (62)
Alt. 3' UTR Intron (33)

Figure S6

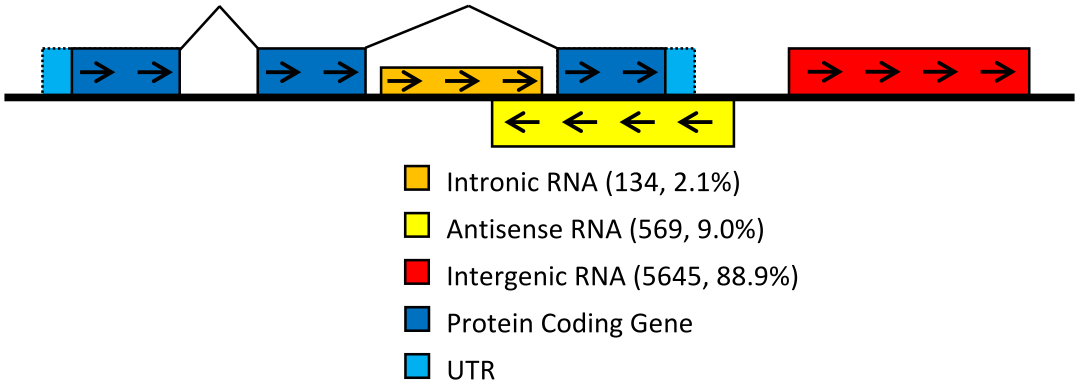

Intronic RNA (134, 2.1%)
Antisense RNA (569, 9.0%)
Intergenic RNA (5645, 88.9%)
Protein Coding Gene
UTR

Figure S7

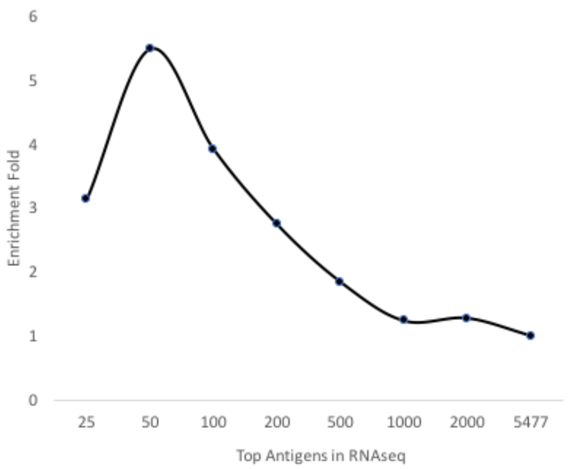

6
5
4
3
2
1
0
Enrichment Fold
25
50
100
200
500
1000
2000
5477
Top Antigens in RNAseq

Figure S8

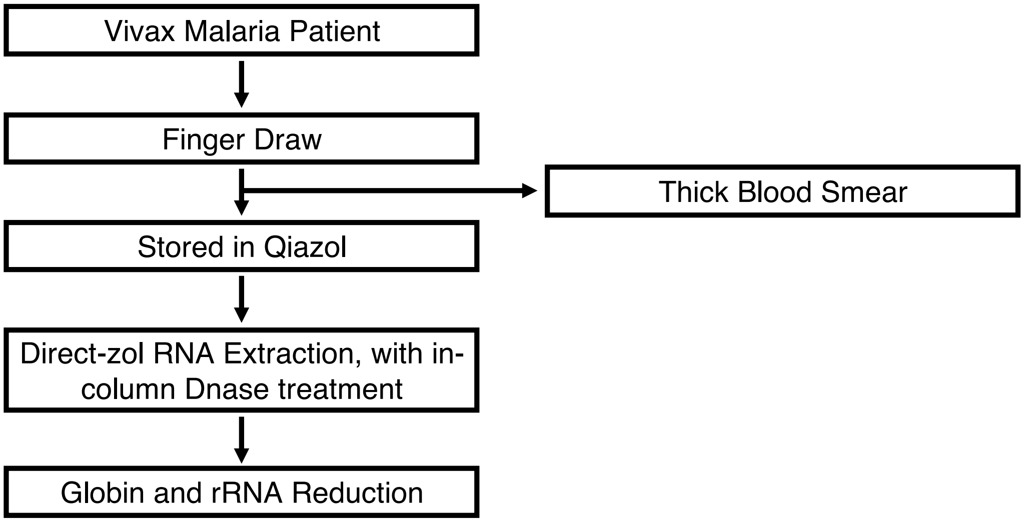
Vivax Malaria Patient
Finger Draw
Thick Blood Smear
Stored in Qiazol
Direct-zol RNA Extraction, with in-column Dnase treatment
Globin and rRNA Reduction

Figure S9

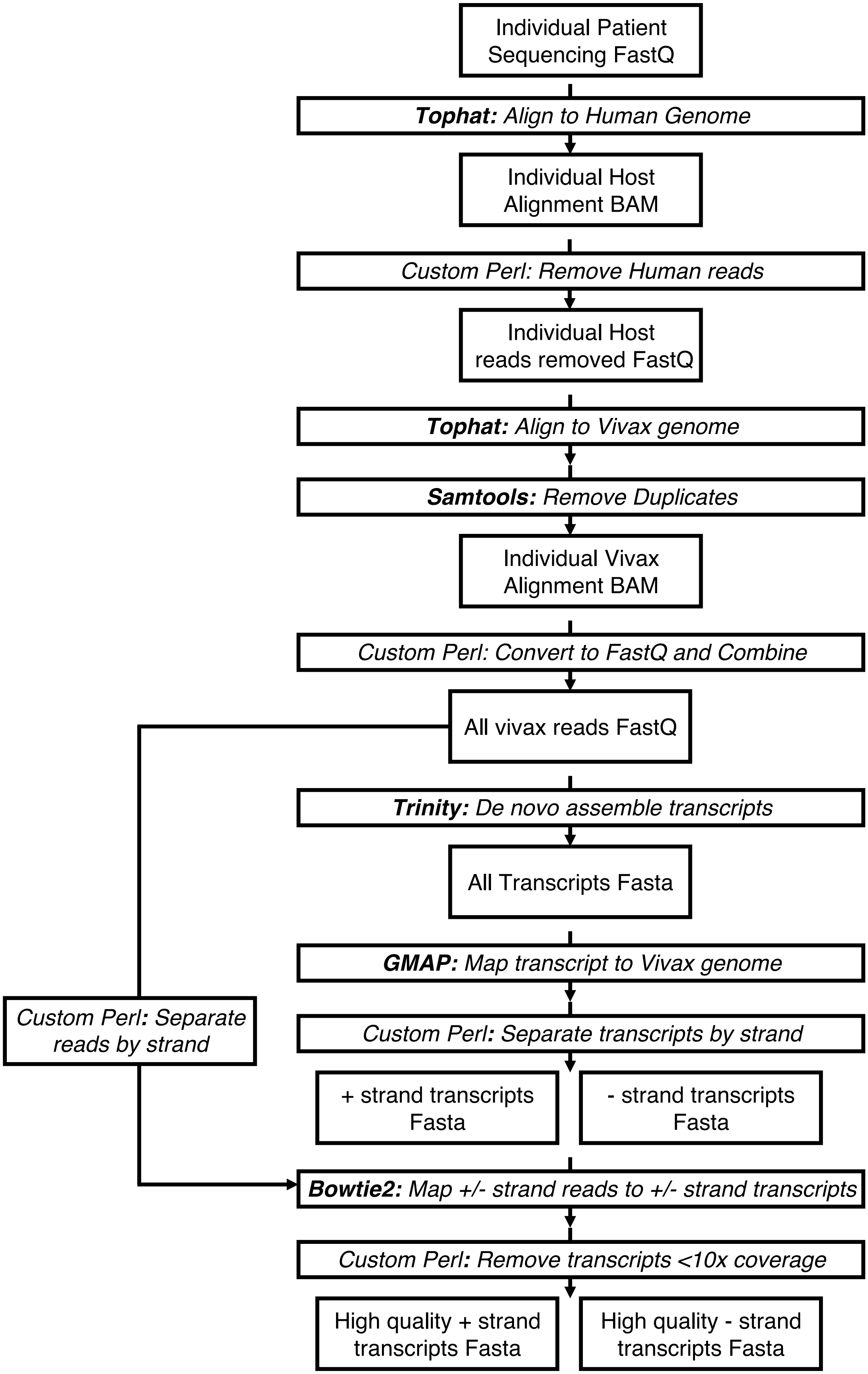

Individual Patient Sequencing FastQ
Tophat: Align to Human Genome
Individual Host Alignment BAM
Custom Perl: Remove Human reads
Individual Host reads removed FastQ
Tophat: Align to Vivax genome
Samtools: Remove Duplicates
Individual Vivax Alignment BAM
Custom Perl: Convert to FastQ and Combine
All vivax reads FastQ
Trinity: De novo assemble transcripts
All Transcripts Fasta
GMAP: Map transcript to Vivax genome
Custom Perl: Separate reads by strand
Custom Perl: Separate transcripts by strand
+ strand transcripts Fasta
- strand transcripts Fasta
Bowtie2: Map +/- strand reads to +/- strand transcripts
Custom Perl: Remove transcripts <10x coverage
High quality + strand transcripts Fasta
High quality - strand transcripts Fasta
